# Correction: Near-infrared spectroscopy of the placenta for monitoring fetal oxygenation during labour

**DOI:** 10.1371/journal.pone.0233830

**Published:** 2020-05-21

**Authors:** The PLOS ONE Staff

There are errors in the panel labels and caption for Fig 3. Please view the correct Fig 3 and caption here.

**Fig 3:**
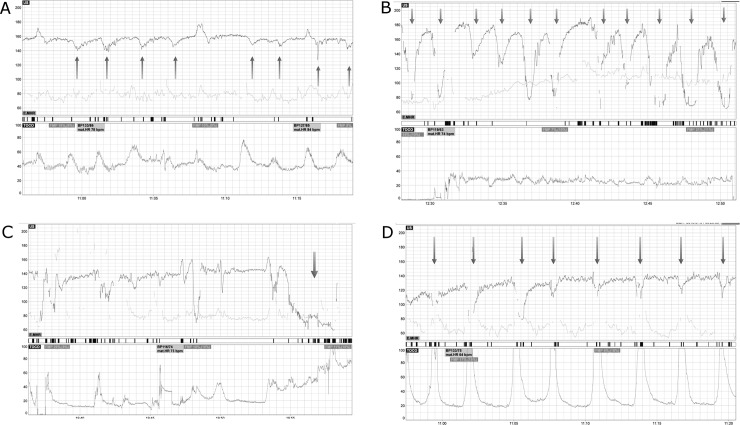
Examples of different FHR deceleration subtypes on CTG. **A)** Late FHR decelerations. **B)** Variable FHR decelerations. **C)** Prolonged FHR deceleration. **D)** Early FHR decelerations.

The publisher apologizes for the errors.
